# 3-Nitro­benzaldehyde thio­semicarbazone

**DOI:** 10.1107/S1600536808042645

**Published:** 2008-12-20

**Authors:** De-Hong Wu, Zhu-Feng Li, You-Hong Zhang

**Affiliations:** a Ordered Matter Science Research Center, College of Chemistry and Chemical Engineering, Southeast University, Nanjing 210096, People’s Republic of China

## Abstract

The mol­ecule of the title compound, C_8_H_8_N_4_O_2_S, adopts an *E* configuration about both the C—N bonds. In the crystal structure, adjacent mol­ecules are linked by inter­molecular N—H⋯S hydrogen-bonding inter­actions, forming chains running parallel to the *b* axis.

## Related literature

For general background to thio­semicarbazone compounds, see: Casas *et al.* (2000 ▶); Tarafder *et al.* (2000 ▶); Deschamps *et al.* (2003 ▶); Liu *et al.* (1999 ▶); Wu *et al.* (2000 ▶). For similar structures, see: Sutton (1965 ▶).
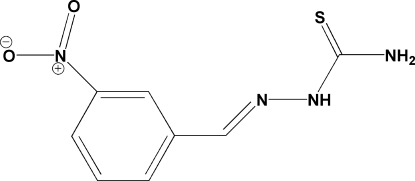


## Experimental

### 

#### Crystal data

C_8_H_8_N_4_O_2_S
*M* *_r_* = 224.25
Monoclinic, 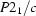

*a* = 13.276 (3) Å
*b* = 8.225 (7) Å
*c* = 10.491 (4) Å
β = 112.78 (5)°
*V* = 1056.2 (11) Å^3^
*Z* = 4
Mo *K*α radiation
μ = 0.29 mm^−1^
*T* = 291 (2) K
0.20 × 0.20 × 0.20 mm

#### Data collection

Rigaku Mercury2 diffractometer
Absorption correction: multi-scan (*CrystalClear*; Rigaku, 2005 ▶) *T*_min_ = 0.930, *T*_max_ = 0.940
9317 measured reflections
2071 independent reflections
1343 reflections with *I* > 2σ(*I*)
*R*_int_ = 0.081

#### Refinement

*R*[*F* ^2^ > 2σ(*F* ^2^)] = 0.069
*wR*(*F* ^2^) = 0.117
*S* = 1.01
2071 reflections
136 parameters
H-atom parameters constrained
Δρ_max_ = 0.23 e Å^−3^
Δρ_min_ = −0.18 e Å^−3^

### 

Data collection: *CrystalClear* (Rigaku, 2005 ▶); cell refinement: *CrystalClear*; data reduction: *CrystalClear*; program(s) used to solve structure: *SHELXS97* (Sheldrick, 2008 ▶); program(s) used to refine structure: *SHELXL97* (Sheldrick, 2008 ▶); molecular graphics: *SHELXTL* (Sheldrick, 2008 ▶); software used to prepare material for publication: *SHELXTL*.

## Supplementary Material

Crystal structure: contains datablocks I, global. DOI: 10.1107/S1600536808042645/rz2279sup1.cif

Structure factors: contains datablocks I. DOI: 10.1107/S1600536808042645/rz2279Isup2.hkl

Additional supplementary materials:  crystallographic information; 3D view; checkCIF report

## Figures and Tables

**Table 1:** Hydrogen-bond geometry (Å, °)

*D*—H⋯*A*	*D*—H	H⋯*A*	*D*⋯*A*	*D*—H⋯*A*
N1—H1*B*⋯S1^i^	0.86	2.56	3.369 (4)	158
N2—H2*B*⋯S1^ii^	0.86	2.56	3.394 (4)	165

Symmetry codes: (i) 
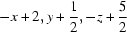
; (ii) 
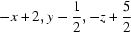
.

**Fig. 1.**
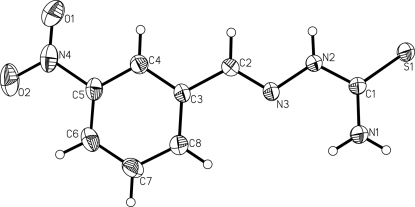
The molecular structure of the title compound, showing the atomic numbering scheme. Displacement ellipsoids are drawn at the 30% probability level.

## supplementary crystallographic information

### Comment

Thiosemicarbazones constitute an important class of N, S donor ligands with their propensity to react with a wide range of metals (Casas *et al.*, 2000). Schiff bases show potential activity as antimicrobial and anticancer agents (Tarafder *et al.*, 2000; Deschamps *et al.*, 2003) and so have biochemical and pharmacological applications. It has been postulated that extensive electron delocalization in the thiosemicarbazone moiety helps the free thiosemicarbazone ligands and their metal complexes to exhibit SHG (second harmonic generation) efficiency (Liu *et al.*, 1999; Wu *et al.*, 2000). As part of a research on non-linear optical materials, specifically thiosemicarbazones and their metal complexes, we report here the crystal structure of a new Schiff base compound derived from thiosemicarbazide and 3-nitrobenzaldehyde.

In the title compound (Fig. 1), the thiosemicarbazone moiety is nearly planar (maximum deviation 0.077 (3) Å for atom N2) and shows an *E* configuration about the C2—N3 bond. The molecule is not strictly planar, the dihedral angle between the thiosemicarbazone moiety and the phenyl ring being 13.45 (12)°. The C—S bond distance of 1.695 (3) Å agrees well with similar bonds in related compounds, being intermediate between 1.82 Å for a C—S single bond and 1.56 Å for a C=S double bond (Sutton, 1965). The C1—N2 bond distance (1.346 (4) Å) is indicative of some double-bond character, suggesting extensive electron delocalization in the whole molecule. In the crystal packing, adjacent molecules are linked by intermolecular N—H···S hydrogen bonds (Table 1) to form chains running parallel to the *b* axis.

### Experimental

The title compound was synthesized by refluxing 3-nitrobenzaldehyde (6.04 g, 4 mmol) and thiosemicarbazide (0.37 g, 4 mmol) in absolute ethanol (30 ml) for 8 h. After cooling to room temperature, the yellow solid formed was isolated and dried under vacuum (0.76 g, yield 85%). Single crystals suitable for X-ray structure analysis were obtained by slow evaporation of an ethanol solution in air.

### Refinement

H atoms were placed at calculated positions (N—H = 0.86 Å, C—H = 0.93 Å), and refined using the riding model approximation, with *U*_iso_(H) = 1.2 *U*_eq_ (C, N).

### Crystal data

**Table:**

C_8_H_8_N_4_O_2_S	*F*(000) = 464
*M**_r_* = 224.25	*D*_x_ = 1.410 Mg m^−^^3^
Monoclinic, *P*2_1_/*c*	Mo *K*α radiation, λ = 0.71073 Å
Hall symbol: -P 2ybc	Cell parameters from 1712 reflections
*a* = 13.276 (3) Å	θ = 3.0–27.4°
*b* = 8.225 (7) Å	µ = 0.29 mm^−^^1^
*c* = 10.491 (4) Å	*T* = 291 K
β = 112.78 (5)°	Block, yellow
*V* = 1056.2 (11) Å^3^	0.20 × 0.20 × 0.20 mm
*Z* = 4	

### Data collection

**Table:**

Rigaku Mercury2 diffractometer	2071 independent reflections
Radiation source: fine-focus sealed tube	1343 reflections with *I* > 2σ(*I*)
graphite	*R*_int_ = 0.081
Detector resolution: 13.6612 pixels mm^-1^	θ_max_ = 26.0°, θ_min_ = 3.0°
CCD_Profile_fitting scans	*h* = −16→16
Absorption correction: multi-scan (*CrystalClear*; Rigaku, 2005)	*k* = −10→10
*T*_min_ = 0.930, *T*_max_ = 0.940	*l* = −12→12
9317 measured reflections	

### Refinement

**Table:**

Refinement on *F*^2^	Primary atom site location: structure-invariant direct methods
Least-squares matrix: full	Secondary atom site location: difference Fourier map
*R*[*F*^2^ > 2σ(*F*^2^)] = 0.069	Hydrogen site location: inferred from neighbouring sites
*wR*(*F*^2^) = 0.117	H-atom parameters constrained
*S* = 1.01	*w* = 1/[σ^2^(*F*_o_^2^) + (0.0122*P*)^2^ + 1.5479*P*] where *P* = (*F*_o_^2^ + 2*F*_c_^2^)/3
2071 reflections	(Δ/σ)_max_ < 0.001
136 parameters	Δρ_max_ = 0.23 e Å^−^^3^
0 restraints	Δρ_min_ = −0.18 e Å^−^^3^

### Special details

**Table:**

Geometry. All e.s.d.'s (except the e.s.d. in the dihedral angle between two l.s. planes) are estimated using the full covariance matrix. The cell e.s.d.'s are taken into account individually in the estimation of e.s.d.'s in distances, angles and torsion angles; correlations between e.s.d.'s in cell parameters are only used when they are defined by crystal symmetry. An approximate (isotropic) treatment of cell e.s.d.'s is used for estimating e.s.d.'s involving l.s. planes.
Refinement. Refinement of *F*^2^ against ALL reflections. The weighted *R*-factor *wR* and goodness of fit *S* are based on *F*^2^, conventional *R*-factors *R* are based on *F*, with *F* set to zero for negative *F*^2^. The threshold expression of *F*^2^ > σ(*F*^2^) is used only for calculating *R*-factors(gt) *etc*. and is not relevant to the choice of reflections for refinement. *R*-factors based on *F*^2^ are statistically about twice as large as those based on *F*, and *R*- factors based on ALL data will be even larger.

### Fractional atomic coordinates and isotropic or equivalent isotropic displacement parameters (Å^2^)

**Table:**

	*x*	*y*	*z*	*U*_iso_*/*U*_eq_	
C1	0.9248 (3)	1.1141 (4)	1.1340 (3)	0.0416 (8)	
C2	0.8132 (3)	0.8365 (4)	0.8634 (3)	0.0444 (9)	
H2A	0.8406	0.7429	0.9149	0.053*	
C3	0.7415 (3)	0.8218 (4)	0.7163 (3)	0.0412 (9)	
C4	0.7111 (3)	0.6666 (4)	0.6615 (3)	0.0441 (9)	
H4A	0.7375	0.5743	0.7153	0.053*	
C5	0.6401 (3)	0.6545 (5)	0.5237 (4)	0.0476 (10)	
C6	0.5984 (3)	0.7864 (5)	0.4403 (4)	0.0584 (11)	
H6A	0.5501	0.7733	0.3490	0.070*	
C7	0.6301 (3)	0.9401 (5)	0.4955 (4)	0.0603 (12)	
H7A	0.6036	1.0314	0.4404	0.072*	
C8	0.7017 (3)	0.9588 (5)	0.6334 (4)	0.0512 (10)	
H8A	0.7229	1.0622	0.6697	0.061*	
N1	0.8999 (3)	1.2497 (4)	1.0619 (3)	0.0572 (10)	
H1A	0.8691	1.2460	0.9732	0.069*	
H1B	0.9145	1.3420	1.1036	0.069*	
N2	0.9000 (2)	0.9732 (3)	1.0633 (3)	0.0425 (8)	
H2B	0.9222	0.8823	1.1055	0.051*	
N3	0.8385 (2)	0.9751 (3)	0.9216 (3)	0.0411 (7)	
N4	0.6100 (3)	0.4887 (5)	0.4659 (4)	0.0656 (10)	
O1	0.6466 (3)	0.3718 (4)	0.5411 (4)	0.0905 (11)	
O2	0.5489 (3)	0.4772 (5)	0.3440 (3)	0.1039 (13)	
S1	0.98388 (9)	1.10945 (12)	1.30902 (9)	0.0529 (3)	

### Atomic displacement parameters (Å^2^)

**Table:**

	*U*^11^	*U*^22^	*U*^33^	*U*^12^	*U*^13^	*U*^23^
C1	0.046 (2)	0.038 (2)	0.0381 (19)	−0.0034 (18)	0.0136 (17)	0.0019 (17)
C2	0.048 (2)	0.041 (2)	0.0373 (19)	0.0029 (18)	0.0093 (17)	0.0012 (17)
C3	0.045 (2)	0.044 (2)	0.0326 (18)	−0.0016 (17)	0.0121 (17)	−0.0025 (16)
C4	0.043 (2)	0.047 (2)	0.041 (2)	0.0013 (17)	0.0138 (18)	−0.0016 (17)
C5	0.038 (2)	0.056 (3)	0.047 (2)	−0.0044 (18)	0.0144 (18)	−0.0142 (19)
C6	0.048 (3)	0.077 (3)	0.040 (2)	0.003 (2)	0.007 (2)	−0.008 (2)
C7	0.063 (3)	0.061 (3)	0.046 (2)	0.016 (2)	0.010 (2)	0.009 (2)
C8	0.054 (3)	0.047 (2)	0.045 (2)	0.0008 (19)	0.0120 (19)	−0.0025 (18)
N1	0.084 (3)	0.0394 (19)	0.0371 (17)	−0.0042 (17)	0.0120 (18)	−0.0012 (14)
N2	0.054 (2)	0.0345 (17)	0.0332 (16)	−0.0005 (14)	0.0102 (15)	−0.0003 (13)
N3	0.0443 (19)	0.0418 (18)	0.0335 (15)	−0.0040 (14)	0.0110 (14)	−0.0023 (13)
N4	0.056 (3)	0.076 (3)	0.063 (3)	−0.017 (2)	0.022 (2)	−0.029 (2)
O1	0.107 (3)	0.056 (2)	0.095 (3)	−0.016 (2)	0.024 (2)	−0.0196 (19)
O2	0.104 (3)	0.110 (3)	0.069 (2)	−0.028 (2)	0.003 (2)	−0.042 (2)
S1	0.0735 (7)	0.0436 (5)	0.0345 (5)	−0.0028 (5)	0.0130 (5)	−0.0035 (4)

### Geometric parameters (Å, °)

**Table:**

C1—N1	1.316 (4)	C6—C7	1.386 (5)
C1—N2	1.346 (4)	C6—H6A	0.9300
C1—S1	1.695 (3)	C7—C8	1.398 (5)
C2—N3	1.276 (4)	C7—H7A	0.9300
C2—C3	1.471 (4)	C8—H8A	0.9300
C2—H2A	0.9300	N1—H1A	0.8600
C3—C4	1.394 (5)	N1—H1B	0.8600
C3—C8	1.396 (5)	N2—N3	1.392 (4)
C4—C5	1.391 (5)	N2—H2B	0.8600
C4—H4A	0.9300	N4—O1	1.219 (5)
C5—C6	1.369 (5)	N4—O2	1.225 (4)
C5—N4	1.483 (5)		
			
N1—C1—N2	117.4 (3)	C7—C6—H6A	120.9
N1—C1—S1	123.3 (3)	C6—C7—C8	120.5 (4)
N2—C1—S1	119.3 (3)	C6—C7—H7A	119.8
N3—C2—C3	121.3 (3)	C8—C7—H7A	119.8
N3—C2—H2A	119.4	C3—C8—C7	119.9 (4)
C3—C2—H2A	119.4	C3—C8—H8A	120.1
C4—C3—C8	120.2 (3)	C7—C8—H8A	120.1
C4—C3—C2	118.3 (3)	C1—N1—H1A	120.0
C8—C3—C2	121.5 (3)	C1—N1—H1B	120.0
C5—C4—C3	117.8 (3)	H1A—N1—H1B	120.0
C5—C4—H4A	121.1	C1—N2—N3	119.7 (3)
C3—C4—H4A	121.1	C1—N2—H2B	120.1
C6—C5—C4	123.5 (4)	N3—N2—H2B	120.1
C6—C5—N4	119.3 (3)	C2—N3—N2	116.0 (3)
C4—C5—N4	117.2 (4)	O1—N4—O2	123.5 (4)
C5—C6—C7	118.3 (4)	O1—N4—C5	118.9 (3)
C5—C6—H6A	120.9	O2—N4—C5	117.6 (4)
			
N3—C2—C3—C4	−175.8 (4)	C2—C3—C8—C7	−177.4 (4)
N3—C2—C3—C8	2.5 (6)	C6—C7—C8—C3	−0.1 (6)
C8—C3—C4—C5	−0.6 (5)	N1—C1—N2—N3	−7.7 (5)
C2—C3—C4—C5	177.7 (3)	S1—C1—N2—N3	171.4 (2)
C3—C4—C5—C6	−0.4 (6)	C3—C2—N3—N2	175.3 (3)
C3—C4—C5—N4	178.9 (3)	C1—N2—N3—C2	−176.1 (3)
C4—C5—C6—C7	1.1 (6)	C6—C5—N4—O1	−179.3 (4)
N4—C5—C6—C7	−178.2 (4)	C4—C5—N4—O1	1.3 (6)
C5—C6—C7—C8	−0.8 (6)	C6—C5—N4—O2	0.5 (6)
C4—C3—C8—C7	0.9 (6)	C4—C5—N4—O2	−178.9 (4)

### Hydrogen-bond geometry (Å, °)

**Table:**

*D*—H···*A*	*D*—H	H···*A*	*D*···*A*	*D*—H···*A*
N1—H1B···S1^i^	0.86	2.56	3.369 (4)	158
N2—H2B···S1^ii^	0.86	2.56	3.394 (4)	165

Symmetry codes: (i) −*x*+2, *y*+1/2, −*z*+5/2; (ii) −*x*+2, *y*−1/2, −*z*+5/2.
